# Correction: Predictive modeling of gene expression and localization of DNA binding site using deep convolutional neural networks

**DOI:** 10.1371/journal.pcbi.1014647

**Published:** 2026-08-11

**Authors:** Arman Karshenas, Tom Röschinger, Hernan G. Garcia

The first author, Arman Karshenas, is incorrectly noted as the corresponding author. The correct corresponding author is Hernan G. Garcia. Hernan G. Garcia’s email address is: hggarcia@berkeley.edu.
