# Supplementary material for: Predictive modeling of gene expression and localization of DNA binding site using deep convolutional neural networks
Source: PLoS Comput Biol. 2026 Apr 1;22(4):e1014092. doi: 10.1371/journal.pcbi.1014092 (PMC13052891; doi:10.1371/journal.pcbi.1014092)
Supplement: S1 Text — (PDF) [file pcbi.1014092.s001.pdf]

Supplementary Information

MPRA Dataset Example

The raw RNA-Seq data from *E. coli* cultures is processed as described in the “RNA-seq Raw Data Processing” section of the Materials and Methods. The processed data is tabulated to form what is referred to as the MPRA dataset throughout this work. Table A shows a few rows of this type of data for the illustrative *yqhC* operon.

Table A. Illustrative example of the differential expression dataset used throughout this work. This table features processed MPRA data for the *yqhC* operon. Each row represents a uniquely mutated 160 bp-long promoter sequence. The dataset includes the following columns: 1) DNA Sequence: The 160 bp-long DNA sequence of the mutated promoter. 2) RNA count: The measured expression level of the reporter gene, as quantified by RNA-Seq. This reflects the transcriptional activity associated with the promoter sequence. 3) DNA count: The count of DNA barcodes corresponding to the copy number of each sequence in the library. This serves as a measure of the copy number of the plasmid containing each regulatory sequence. 4) $\log\left(\frac{\text{RNA count}}{\text{DNA count}}\right)$: A normalized measure of expression, calculated by dividing the RNA count by the DNA count and taking the logarithm of the result. This normalization accounts for variations in sequence abundance and enables direct comparison of transcriptional activities across sequences. 5) Label: A discretized classification assigned to each sequence, derived from binning the normalized log-expression values into categories based on a binning algorithm (1: zero expression bin, 2: low expression bin, 3: high expression bin) as described in the “RNA Count Labeling” section of the Materials and Methods. This table format is applied consistently across all operons analyzed in this study, allowing for a systematic comparison of promoter sequence variants and their transcriptional activities.

DNA sequence	RNA count	DNA count	$\log\left(\frac{\text{RNA count}}{\text{DNA count}}\right)$	label
CTGCGCAGATTACAGTTGTTCATTACTTCC...	64	1	4.16	3
GTCTGCAGCGTAAACTCGTTCATGACTTGG...	3	36	-2.48	2
CGGTGCAGATTATAGATGTTCATTTCATGC...	5	7	-0.34	3
GTGTGCACATTAAAGTTGTTCATTACTTGC...	1	33	-3.50	2
GTGTGCAGTTGAAAGTTGTTCATTCCTTGA...	3	14	-1.54	2
