# Supplementary material for: Predictive modeling of gene expression and localization of DNA binding site using deep convolutional neural networks
Source: PLoS Comput Biol. 2026 Apr 1;22(4):e1014092. doi: 10.1371/journal.pcbi.1014092 (PMC13052891; doi:10.1371/journal.pcbi.1014092)
Supplement: S2 Text — (PDF) [file pcbi.1014092.s002.pdf]

Supplementary Information

Comparing DARSI to Other Computational Approaches

The last few years have seen remarkable progress in the application of advanced machine learning architectures—including transformer-based models and large-scale pretrained foundational models—to problems in regulatory genomics. In this section, we provide additional details on the relative merits and limitations of these approaches compared to DARSI, as well as the rationale for our decision to adopt a convolutional neural network (CNN)-based framework.

Several deep learning architectures have been proposed for modeling regulatory sequences, including Enformer (manuscript reference [34]), BPNet (manuscript reference [33]), Basenji2 (manuscript reference [30]), and various pretrained transformer models such as DNABERT (manuscript reference [37]) and the Nucleotide Transformer (manuscript reference [38]). These methods have demonstrated substantial predictive power in large, diverse genomic datasets, measured by the accuracy of downstream tasks such as promoter region classification (manuscript reference [38]), and have been applied to a variety of downstream tasks, such as promoter classification, splice site detection, and gene expression prediction.

However, their applicability to MPRA-derived datasets—particularly those with limited sample size—presents several practical and methodological challenges. First, transformer-based architectures typically require very large volumes of training data to achieve optimal performance. For example, the Nucleotide Transformer was pretrained on approximately 2×10^{11} nucleotides and contains hundreds of millions to billions of parameters (manuscript reference [38]). Even fine-tuning these models on a specific task often requires tens of thousands of labeled sequences to avoid overfitting and instability (1). In contrast, our MPRA experiments yield approximately 1,000 variants per operon (corresponding to $\sim 160,000$ nucleotides in total), which is orders of magnitude smaller than the training datasets used in foundational model studies.

Moreover, while embeddings—vector representations of the input sequences that capture patterns in nucleotide sequences learned during training—generated by pretrained models can be used as input features for downstream classifiers, their effectiveness depends on how closely the data used to pretrain the model resembles the sequences encountered in the task at hand. Most foundational models are trained on a mixture of genomes from both bacterial and eukaryotic organisms (manuscript references [37,38]), meaning that their representations may emphasize sequence patterns that are not relevant—or are less prominent—in bacterial operon regulatory regions. In cases where the biological context of the target task diverges significantly from the training distribution, performance can degrade unless the model is fine-tuned on a representative dataset. However, fine-tuning these models requires large numbers of labeled examples, which are typically unavailable in MPRA datasets due to experimental and cost constraints. For example, models like DNABERT or the Nucleotide Transformer require tens of thousands of samples to fine-tune effectively for downstream classification tasks (manuscript references [37,38]). These considerations limit the applicability of pretrained transformer embeddings in small-scale, context-specific tasks such as predicting expression outcomes from bacterial MPRA data.

Another important consideration is interpretability. A major goal of this study was not only to predict gene expression levels from sequence but also to identify putative regulatory binding sites in a manner that is biologically interpretable. Transformer-based models rely on tokenized representations of the input sequence. This tokenization is typically implemented by splitting the input sequence into overlapping or non-overlapping short segments called k-mers For example, a sequence. "ACGTGCTA" could be broken into overlapping 6-mers like "ACGTGC," "CGTGCT," and

"GTGCTA". Instead of analyzing individual nucleotides, the model processes these k-mer tokens. As a result, this tokenization makes it difficult to directly map model predictions or importance scores back to specific base pair positions in the original DNA sequence, reducing biological interpretability. Furthermore, these models use multiple layers of self-attention and nonlinear transformations to generate embeddings which, in this case of tokenization, correspond to high-dimensional vector representations of the input tokens. Because these embeddings do not have a direct one-to-one correspondence with nucleotide positions, and because outputs may involve complex interactions between distant tokens, generating saliency maps or other gradient-based attributions becomes both computationally demanding and methodologically ambiguous (2; 3).

By contrast, convolutional neural networks (CNNs) operate directly on the one-hot encoded nucleotide matrix and preserve the spatial alignment between input and learned features across layers. This architectural property allows for direct backpropagation of gradients from the output prediction to each input base, making saliency analysis more tractable and interpretable. As a result, CNNs enable us to identify nucleotide-level contributions to gene expression predictions with greater clarity, a feature that was central to our approach for localizing candidate regulatory binding sites.

Finally, the computational cost of training or fine-tuning large transformer architectures significantly exceeds that of CNN-based models. Transformer models contain hundreds of millions to billions of parameters and require substantial memory bandwidth, often necessitating high-memory GPUs or TPU clusters to support even modest-scale adaptation. For instance, fine-tuning models like DNABERT or the Nucleotide Transformer has been reported to require multiple A100 GPUs or equivalent infrastructure (manuscript references [37,38], (4)). In contrast, DARSI was designed to be efficiently trainable on a single GPU with modest compute resources, which facilitates broader accessibility and improves reproducibility across laboratories with limited hardware availability.

As a result, while our choice of a CNN-based approach may not achieve the absolute maximal predictive performance reported by foundational models on large benchmark datasets, it offers a practical balance between model capacity, interpretability, and compatibility with MPRA experiments. The resulting framework allows accurate prediction of discretized gene expression levels and systematic identification of regulatory regions with limited data requirements, consistent with the main objectives of this study. Future work may explore the feasibility of hybrid approaches that combine pretrained embeddings with convolutional classifiers, or of scaling MPRA experiments to enable fine-tuning of more complex architectures. Nonetheless, in the context of our data and objectives, DARSI provides an effective and tractable solution to regulatory sequence mapping.

References

[1] Zambrano Chaves, J.M., Wang, E., Tu, T., Vaishnav, E.D., Lee, B., Mahdavi, S.S., Semturs, C., Fleet, D., Natarajan, V., and Azizi, S. (2024). Tx-LLM: A Large Language Model for Therapeutics. *arXiv*, 2406.06316.

[2] Chefer, H., Gur, S., and Wolf, L. (2021). Transformer Interpretability Beyond Attention Visualization. *arXiv*, 2012.09838.

[3] Jain, S. and Wallace, B.C. (2019). Attention is not Explanation. *arXiv*, 1902.10186.

[4] Tay, Y., Dehghani, M., Bahri, D., and Metzler, D. (2020). Efficient Transformers: A Survey. *arXiv*, 2009.06732.
