# Supplementary material for: Predictive modeling of gene expression and localization of DNA binding site using deep convolutional neural networks
Source: PLoS Comput Biol. 2026 Apr 1;22(4):e1014092. doi: 10.1371/journal.pcbi.1014092 (PMC13052891; doi:10.1371/journal.pcbi.1014092)
Supplement: S3 Text — (PDF) [file pcbi.1014092.s003.pdf]

Supplementary Information

DARSI Architecture and Training

The convolutional layers used in DARSI have filters spanning a 5-bp range to capture local sequence patterns and nucleotide interactions. While these filters operate on short sequence windows, stacking multiple convolutional layers extends the effective receptive field, allowing the network to model higher-order interactions and long-range dependencies across the regulatory sequences. In principle, this architecture enables DARSI to detect complex regulatory features, such as distant transcription factor binding site interactions. However, the inclusion of additional convolutional layers increases model complexity, which can lead to overfitting.

To determine the optimal architecture, we utilized data from the 10 operons with the largest number of variants (*leuABCD*, *rumB*, *zupT*, *yncD*, *uvrD*, *mscK*, *ftsK*, *yqhC*, *groSL*, and *xylA*). We systematically varied the number of convolutional layers and the number of filters within each layer, evaluating training and validation accuracy to balance model complexity and generalization. Fig AA depicts the changes in training and validation accuracies as the number of convolutional layers increased from 2 to 6, with each layer comprising 16 channels. The results indicate that, while training accuracy increases monotonically, validation accuracy decreases monotonically, signaling overfitting. This suggests that the optimal number of layers lies between 2 and 3. To mitigate potential overfitting when training the network on smaller datasets for other operons, we selected 2 as the optimal number of layers.

With the number of layers fixed, we further examined the impact of the number of channels per layer on training and validation accuracies. Fig AB illustrates the average training and validation accuracies across the same 10 operons as the number of channels per layer is varied. The optimal number of channels was determined to be 32, as this corresponds to the maximum validation accuracy. Thus, the final architecture we converged on, with 2 convolutional layers and 32 filter counts, achieved comparable training and validation accuracies, minimizing overfitting while preserving predictive power.

The optimal architecture for DARSI network comprises 12 hidden layers and was designed, trained, and evaluated using the *Matlab* Deep Learning Toolbox (manuscript reference [47]). The network was trained using stochastic gradient descent with an initial learning rate of 0.001 (manuscript references [48,49], (1)). Training was performed for a maximum of 20 epochs with a mini-batch size of 32, and the learning rate was reduced by 20% every 5 epochs. The training dataset was shuffled at the start of each epoch to improve generalization. Table A outlines the layers of the optimized architecture, along with their descriptions, dimensions, and learnable parameters.

References

[1] Ruder, S. (2017). An overview of gradient descent optimization algorithms. *arXiv*, 1609.04747.

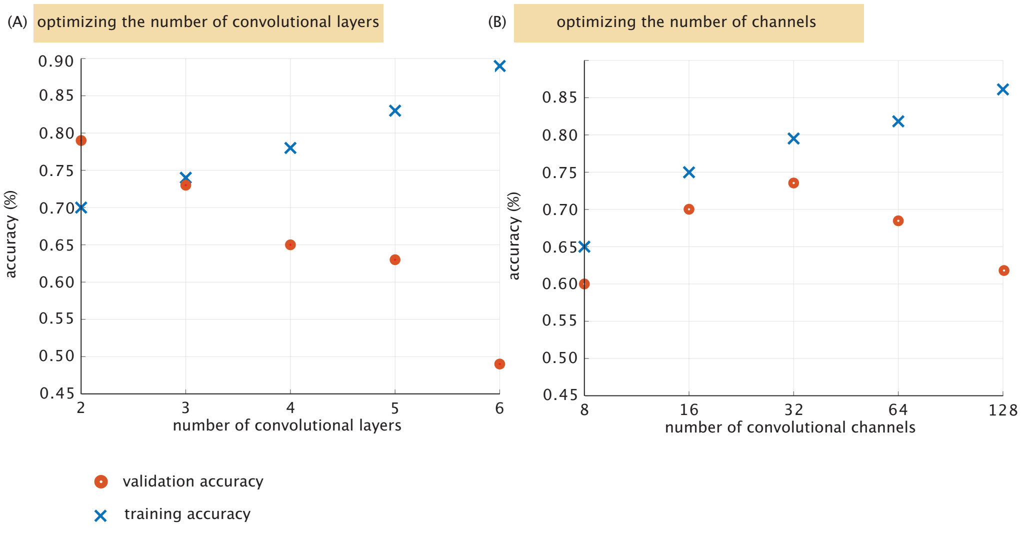

Fig A. DARSI network architecture optimization. (A) The average training and validation accuracy for the 10 operons used for network optimization (*leuABCD, rumB, zupT, yncD, uvrD, mscK, ftsK, yqhC, groSL* and *xylA*) are plotted against the number of convolutional layers. **(B)** Given an optimum of 2 convolutional layers, we assay network training and validation accuracy as a function of the number of channels within each convolutional layer.

Table A. Detailed description of all 12 layers of the optimized DARSI architecture along with their description, dimensions, and number of learnable parameters

Layer name	Type	Dimension	Learnable properties	Number of learnable parameters
Sequence Input	Image input	4x160x1x1	-	0
Conv_1	Convolution	4x5x1x32<br>stride [1 1]<br>padding same	Weights: 4x5x1x32<br>Biases: 1x1x32	672
Batchnorm_1	Batch Normalization	32 channels	Offset: 1x1x32<br>Scale: 1x1x32	64
Relu_1	ReLU	N/A	-	0
Maxpool_1	Max Pooling	1×2<br>stride [2 2]<br>padding [0 0 0 0]	-	0
Conv_2	Convolution	1x5x32x64<br>stride [1 1]<br>padding same	Weights: 1x5x32x64<br>Biases: 1x1x64	10,304
Batchnorm_2	Batch Normalization	64 channels	Offset: 1x1x64<br>Scale: 1x1x64	128
Relu_2	ReLU	N/A	-	0
Maxpool_2	Max Pooling	1x2<br>stride [2 2]<br>padding [0 0 0 0]	-	0
Fc	Fully Connected	1x1x3	Weights: 3x1<br>Biases: 3x1	6
Softmax	Softmax	1x1x3	-	0
Classoutput	Classification Output	1x1x3	-	0
