# Supplementary material for: Predictive modeling of gene expression and localization of DNA binding site using deep convolutional neural networks
Source: PLoS Comput Biol. 2026 Apr 1;22(4):e1014092. doi: 10.1371/journal.pcbi.1014092 (PMC13052891; doi:10.1371/journal.pcbi.1014092)
Supplement: S4 Text — (PDF) [file pcbi.1014092.s004.pdf]

Supplementary Information

Regression vs Classification for Predicting Gene Expression

In this study, we initially explored whether regression models could be used to predict continuous log-normalized gene expression levels from MPRA-derived sequences, before deciding to adopt a discretized classification approach. Below, we describe our rationale for this choice, including empirical observations about the data distribution and theoretical considerations regarding data requirements.

The distribution of expression readouts across the MPRA libraries was highly non-uniform and exhibited multi-modal characteristics. As shown in Fig 2C, the log-transformed expression counts displayed two distinct peaks separated by low-density regions. Such multi-modal structure indicates that the expression landscape is composed of discrete subpopulations rather than a continuous spectrum. In regression tasks, this sparsity of observations between modes increases the effective variance of prediction errors and leads to unstable performance when attempting to interpolate in under-sampled regions (1; 2).

A further complicating factor is the strong imbalance of observations: the majority of sequence variants resulted in no measurable expression, while a minority spanned a broad dynamic range of nonzero expression. Standard techniques for correcting imbalance, such as weighting or subsampling (manuscript references [45,46]), are not directly applicable in regression, since the zero-expression observations encode essential information about disruptive mutations.

To illustrate the impact of these factors on data requirements, it is instructive to consider the expected error scaling under both regression and classification regimes. Let N denote the number of independent and identically distributed observations (sequence variants), σ^2 the variance of measurement noise or intrinsic variability, and k the number of classes in a discretized classification scheme. In a regression setting, the prediction error (for actual observation y and predicted observation $\hat{y}$) variance scales approximately as

$$\mathbb{E}\left[\left(\hat{y}(x) - y(x)\right)^2\right] \sim \frac{\sigma^2}{N}, \tag{S1}$$

but when the target distribution spans several orders of magnitude and includes sparsely populated regions, the effective variance σ^2 increases substantially (3). In practice, for dynamic ranges exceeding 3 logarithmic units, the variance can exceed 1.0, necessitating substantially larger training sets to achieve acceptable error levels.

By contrast, classification error under uniform sampling and balanced classes scales approximately as (2; 1)

$$\mathbb{E}\left[\mathbb{I}\{\hat{y}(x) \neq y(x)\}\right] \sim \frac{k-1}{k} \cdot \frac{1}{N}. \tag{S2}$$

where $\mathbb{I}\{\hat{y}(x) \neq y(x)\}$ is the indicator function, which equals 1 if the predicted label $\hat{y}(x)$ does not match the true label $y(x)$, and 0 otherwise. This function serves as the classification analog to the squared error used in regression. For example, in a 3-class scenario ($k = 3$), and assuming roughly balanced sampling of each bin, the effective number of required observations can be estimated as

$$N_{\text{classification}} \approx \frac{k}{k-1} \cdot N_{\text{per class}}.$$

$N_{\text{per class}}$ is approximately 500–$1,000$, this yields:

$$N_{\text{classification}} \approx \frac{3}{2} \times 1,000 = 1,500.$$

In contrast, regression over the same dynamic range, assuming variance $\sigma^2 \approx 1$, could require $N_{\text{regression}}$ in the range of $10,000$ or more observations to achieve comparable error bounds, reflecting a roughly $6\times$–$7\times$ higher sample complexity (2; 1).

To empirically benchmark regression performance, we trained convolutional regression models on the ten operons with the largest number of sequence variants (*leuABCD*, *rumB*, *zupT*, *yncD*, *uvrD*, *mscK*, *ftsK*, *yqhC*, *groSL*, and *xylA*). Fig A shows the prediction performance of regression models for these operons. Specifically, we report the coefficient of determination (R^2) and the normalized root mean squared error (NRMSE) scaled by the mean expression for each operon. Across this subset, regression models achieved an average R^2 value of 0.06 and an average normalized RMSE of 1.02, indicating limited predictive power relative to the discretized classification approach presented in the main text. Even for operons with the highest number of variants, regression underperformed classification, supporting our decision to adopt a classification framework.

Given the practical constraints of MPRA library sizes (~1,000–2,000 variants per operon), the discrete multi-modal character of the output distribution, and the need to identify salient sequence features without overfitting sparsely populated expression levels, we concluded that a discretized classification approach was more appropriate for the current study. Future work with larger-scale mutagenesis libraries and more continuous expression measurements may revisit regression models as an alternative strategy.

References

[1] Hastie, T., Tibshirani, R., and Friedman, J. (2009). *The Elements of Statistical Learning*. Springer, 2nd edition.

[2] James, G., Witten, D., Hastie, T., and Tibshirani, R. (2013). *An Introduction to Statistical Learning*. Springer.

[3] Bishop, C.M. (2006). *Pattern Recognition and Machine Learning*. Springer.

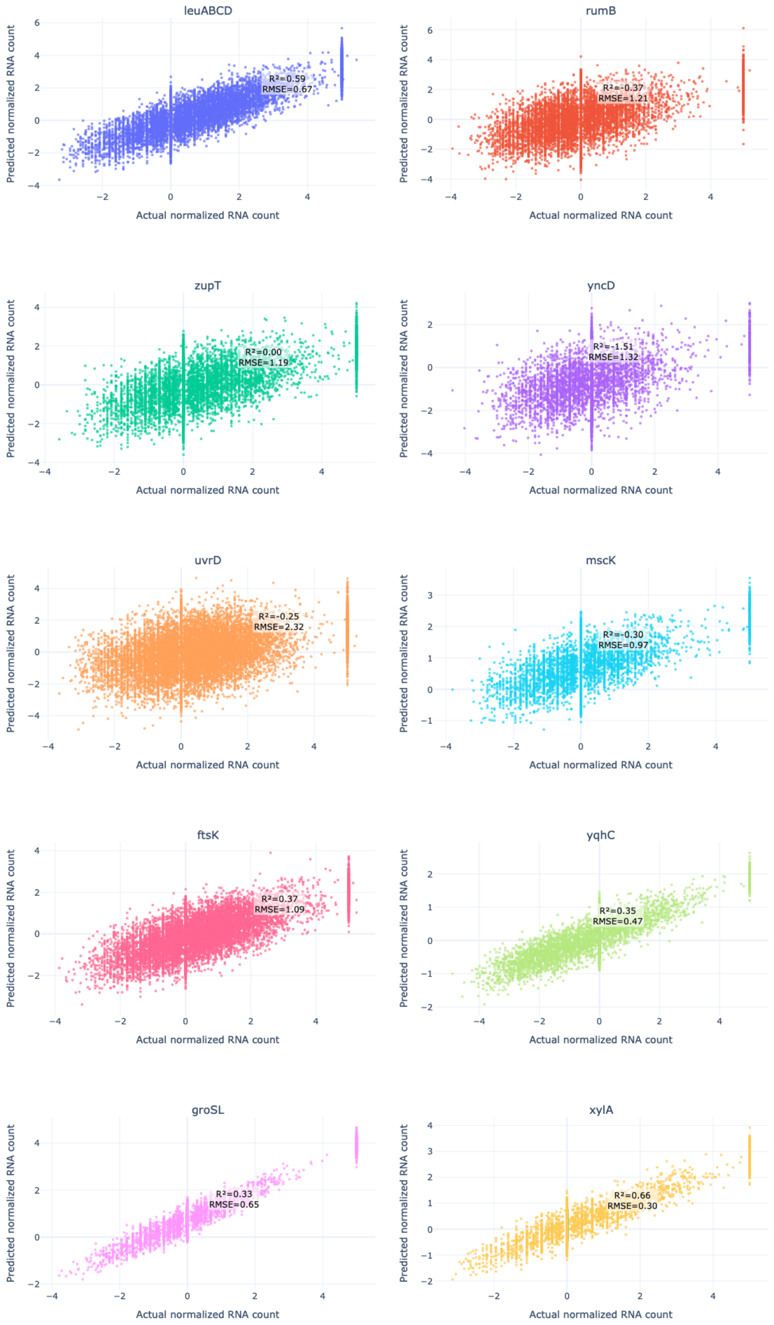

Fig A. Regression model performance on the ten operons with the largest number of sequence variants. Each panel shows predicted versus observed log-normalized expression counts for one operon. The mean R^2 and normalized RMSE values.
