# Supplementary material for: Predictive modeling of gene expression and localization of DNA binding site using deep convolutional neural networks
Source: PLoS Comput Biol. 2026 Apr 1;22(4):e1014092. doi: 10.1371/journal.pcbi.1014092 (PMC13052891; doi:10.1371/journal.pcbi.1014092)
Supplement: S5 Text — (PDF) [file pcbi.1014092.s005.pdf]

Supplementary Information

Performance of a Single CNN Trained Across All Operons

To investigate whether a single convolutional neural network could generalize across multiple operons, we trained a model using all MPRA sequence variants pooled together from all 95 operons. Each input sequence was encoded as a 4×160 binary matrix using the same one-hot encoding scheme described in the main text, and the output was the discretized gene expression bin corresponding to that sequence variant.

The combined dataset comprised approximately 200,000 sequence variants, providing a substantially larger total number of examples compared to training separate models per operon. The same convolutional architecture optimized for individual operon prediction (see S3 Text) was applied here without modification. Training, validation, and testing splits were set to 70%, 15%, and 15%, respectively.

Despite the increased data volume, the predictive performance of this single model was low (Fig A) and comparable to a random classifier that assigns equal probability to each of the three expression bins. Specifically, the average prediction accuracy of the single CNN on the test subset of the 10 operons with the largest number of variants was 34.2%, while a random classifier achieves approximately 33% accuracy. This result indicates that the single model effectively failed to learn meaningful regulatory patterns across operons.

We hypothesize that this lack of predictive power results from the heterogeneity of regulatory architectures across operons: each operon is controlled by a distinct combination of transcription factors with unique numbers, affinities, and positional arrangements of binding sites. Pooling all data likely introduces conflicting sequence–expression relationships that obscure operon-specific signals and reduce model accuracy (manuscript reference [32]; (1)).

These findings confirm that training dedicated operon-specific models provides substantially better predictive power, justifying the modeling strategy used throughout this study.

References

[1] Bishop, C.M. (2006). *Pattern Recognition and Machine Learning*. Springer.

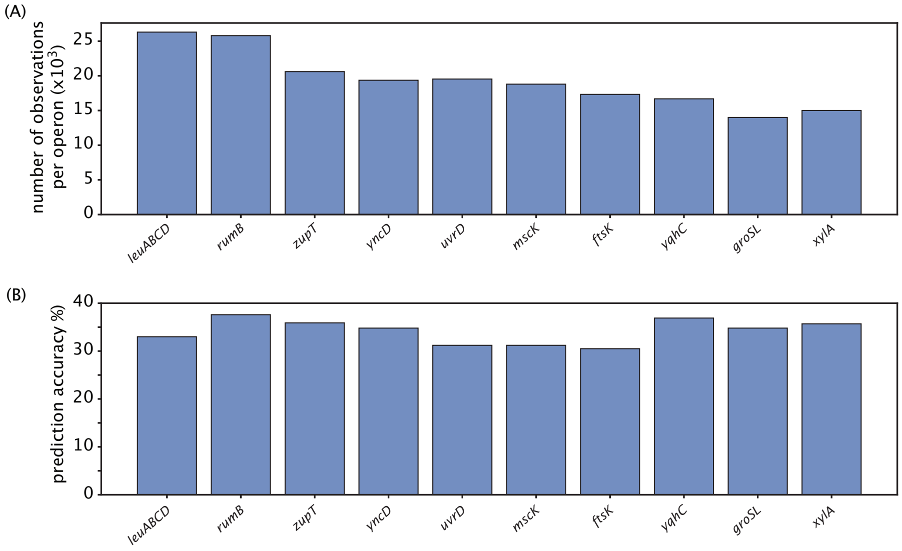

Fig A. Number of observations and prediction accuracy for a single convolutional neural network trained across all operons. (A) Total number of observations available for each of the ten operons with the largest number of variants. **(B)** Prediction accuracy of a single model, trained across all operons, when evaluated on the test set (comprising 15% of the total data) for each operon. This combined approach yields accuracy values comparable to random classification and consistently lower than models trained separately for each operon.
