# Supplementary material for: Predictive modeling of gene expression and localization of DNA binding site using deep convolutional neural networks
Source: PLoS Comput Biol. 2026 Apr 1;22(4):e1014092. doi: 10.1371/journal.pcbi.1014092 (PMC13052891; doi:10.1371/journal.pcbi.1014092)
Supplement: S6 Text — (PDF) [file pcbi.1014092.s006.pdf]

Supplementary Information

Foundational Model Predictive Power

Recent years have seen the emergence of large-scale nucleotide language models trained on massive genomic sequence corpora using self-supervised learning. These foundational models, inspired by advances in natural language processing, are designed to learn general-purpose representations of DNA by predicting masked or contextualized sequence elements across billions of nucleotides spanning diverse organisms. Through this process, the models capture statistical structure, sequence motifs, and higher-order dependencies that reflect functional and evolutionary constraints, without being explicitly trained on any specific downstream task. As a result, embeddings derived from these models have been proposed as a universal feature representation that can be applied to diverse genomic prediction problems, including regulatory element identification, variant effect prediction, and gene expression modeling.

To assess whether such general-purpose nucleotide representations are sufficient to predict MPRA-measured regulatory activity—and to directly compare their performance against DARSI-based architectures optimized for this task—we performed a zero-shot benchmarking analysis using pretrained nucleotide foundation models. Specifically, we evaluated whether embeddings derived from a foundational model—without any task-specific fine-tuning—could predict MPRA-derived gene expression bins from raw DNA sequence. To put this in other words, we attempted to determine the performance of foundational models to perform a task they were not explicitly trained for: classifying sequences into different expression bins as DARSI does. Among available architectures, we selected the Nucleotide Transformer multi-species model (manuscript reference [38]), which is explicitly trained for nucleotide-level representation learning and provides publicly available, stable inference workflows. Alternative models such as EVO (manuscript reference [72]) and EVO-2 (1) were also considered. However, EVO-1 is not natively optimized for raw DNA sequence embedding. Instead, EVO-1 was developed to model higher-level biological properties—such as protein structure, functional constraints, and long-range evolutionary patterns—rather than to capture fine-grained nucleotide-level effects of single-base perturbations in regulatory DNA, which are central to MPRA-based analyses. Further, EVO-2 currently requires a complex GPU software stack that proved incompatible across the multiple systems available in our laboratory. As a result, Nucleotide Transformer constituted the most technically feasible and biologically appropriate foundation model for a controlled zero-shot comparison.

For this analysis, we used the Nucleotide Transformer v2 50M multi-species model to generate embeddings from the fixed-length 160 bp regulatory sequences across all operons in the bacterial MPRA dataset, effectively resulting in a high-dimensional vector representation per sequence. Using these embeddings as fixed features, we trained a simple multi-class logistic regression classifier to assign sequences into three discrete expression bins (zero, low, high). Importantly, we kept the Nucleotide Transformer model weights fixed, so performance reflects the information already encoded in the pretrained representations rather than learning from the MPRA data. Classification was performed independently for each operon using standard train/validation/test splits, mirroring the evaluation protocol used for DARSI-based models.

Across all 95 operons, the Nucleotide Transformer-based zero-shot approach achieved a mean test-set classification accuracy of 48.5%, substantially lower than the 79.8% accuracy obtained by DARSI-trained models on the same task - see Fig A. This ∼30% absolute performance gap demonstrates that, despite their scale and pre-training on orders of magnitude more data, foundational nucleotide language models do not match the predictive power of lightweight, task-aligned architectures when applied in a zero-shot setting to MPRA expression prediction. Full implementation

details—including embedding generation, per-gene classification, and evaluation scripts—are provided in the accompanying GitHub repository to ensure reproducibility. Together, these results indicate that DARSI models achieve superior performance with substantially lower computational and data requirements, highlighting the value of task-specific inductive bias over generic sequence representations for MPRA analysis.

References

[1] Durrant, M.G. et al. (2025). EVO-2: Long-context modeling of genome sequences. *bioRxiv*.

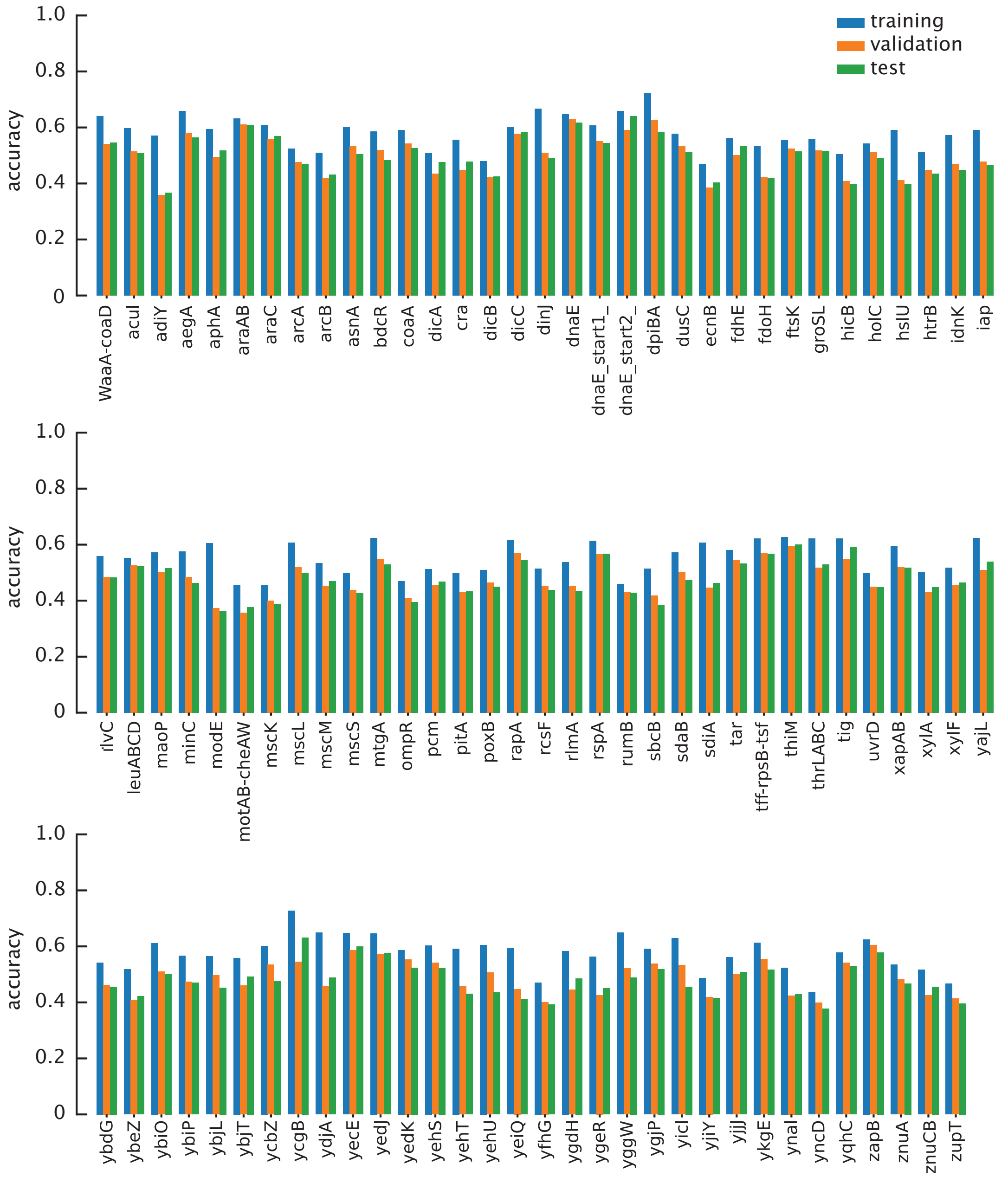

Fig A. Zero-shot MPRA expression prediction using Nucleotide Transformer embeddings. Classification accuracy across all 95 operons using embeddings generated from the pretrained Nucleotide Transformer v2 50M multi-species model without task-specific fine-tuning. For each operon, fixed sequence embeddings were used as input features to train a logistic regression classifier to predict discrete expression bins (zero, low, high). Each point represents test-set accuracy for a single operon under standard train/validation/test splits. The mean accuracy across all operons was 48.5%, substantially lower than the performance achieved by DARSI-based models (79.8%), demonstrating that generic pretrained nucleotide representations alone are insufficient to capture the regulatory effects governing MPRA expression variation.
