# Supplementary material for: Predictive modeling of gene expression and localization of DNA binding site using deep convolutional neural networks
Source: PLoS Comput Biol. 2026 Apr 1;22(4):e1014092. doi: 10.1371/journal.pcbi.1014092 (PMC13052891; doi:10.1371/journal.pcbi.1014092)
Supplement: S7 Text — (PDF) [file pcbi.1014092.s007.pdf]

Supplementary Information

Comparing the DARSI and Mutual Information Approaches

Most peaks in DARSI saliency plots correspond to regions of high mutual information reported by (Ireland et al. (2020)), affirming the capacity of DARSI to detect key regulatory elements. S2 Fig highlights peaks for activating and repressing regions that show strong overlap between the two methods. Specifically, in S2 Fig A we show how, for the *dpiBA* operon, DARSI identifies a clear peak which aligns with a region of high mutual information.

While DARSI and mutual information footprints share similarities, there are notable differences between these two measures. For example, in S2 Fig B we present a comparison between DARSI and mutual information for the *coaA* operon. The figure shows that, while there is a clear correspondence between peaks reported by DARSI and mutual information, a relative shift of the peaks can be observed. We speculate that these slight positional shifts can occur because the convolutional layers output values are processed by a maximum pooling layer. This layer selects the highest value within a 2 bp window, effectively averaging the signal over small regions and potentially shifting windows by 1 or 2 bp.

The differences between DARSI and mutual information also become obvious in the context of the *yqhC* operon shown in S2 Fig C. The figure shows how while some peaks are only identified through DARSI, some other peaks are only present through the mutual information description. Comparative plots, similar to S2 Fig, for all 95 operons in this study can be found in the GitHub repository.

Overall, S2 Fig suggests that peaks generated by DARSI tend to be broader, which may reflect either a biologically meaningful characteristic—such as broader peaks capturing actual regulatory sites—or a consequence of information diffusion through the network's convolutional layers. Further, DARSI identifies more continuous regions of activation and repression, characterized by smooth and extended stretches of blue or red bars in the saliency plots, whereas mutual information plots often exhibit scattered, discrete regions of activity. These differences may reflect the assumption of independence between base pairs underlying mutual information analysis, or potential overfitting in DARSI's predictions.

Importantly, given the complexity of the DARSI architecture—and as the case with most neural networks—dissecting the inner workings of the network to explain the observed differences between its outputs and those of conventional methods remains highly challenging and speculative. Indeed, because of the ultimate "black box" nature of DARSI, an important limitation of the saliency maps generated with this network is their lack of direct physical interpretation: they are unitless in contrast to the interpretable, information-theoretic units provided by mutual information (bits). Despite these drawbacks with interpretability, DARSI's ability to incorporate nucleotide interactions offers a complementary perspective that extends beyond the scope of traditional methods.
