# Supplementary material for: Predictive modeling of gene expression and localization of DNA binding site using deep convolutional neural networks
Source: PLoS Comput Biol. 2026 Apr 1;22(4):e1014092. doi: 10.1371/journal.pcbi.1014092 (PMC13052891; doi:10.1371/journal.pcbi.1014092)
Supplement: S8 Text — (PDF) [file pcbi.1014092.s008.pdf]

Supplementary Information

Example of Saliency Map Computation Using Backpropagation

In neural networks, computing the derivative of the output with respect to the input involves propagating gradients backward through the network—a process known as backpropagation. This task becomes increasingly complex as the number of hidden layers grows and the architecture incorporates advanced components such as convolutional, pooling, and activation layers. To provide clarity and intuition about this process, we include a toy example in this section, illustrating how backpropagation operates specifically through convolutional layers. This example is intended to demystify the mechanics of gradient computation and offer a simplified yet instructive view of how saliency maps are generated.

Consider the sequence shown on the left of Fig AA. To compute the derivative of the network's loss function, $L(\vec{P}) : \mathbb{R}^3 \rightarrow \mathbb{R}$, with respect to each position in the input sequence x_i, we use the cross-entropy loss function, defined as

$$L(\vec{p}, \vec{y}) = \sum_{i=1}^{3} -y_i \log p_i = -(y_1 \log p_1 + y_2 \log p_2 + y_3 \log p_3), \tag{S1}$$

where p_i represents the probability that the sequence belongs to expression bin i, and $\vec{y} = \{y_1, y_2, y_3\}$ is a binary one-hot encoded vector indicating the ground truth class. In $\vec{y}$, only one element is 1, corresponding to the correct class, while the rest are 0. For this example, assume the sequence belongs to the low-expression class. This results in a ground truth vector of $\vec{y} = \{0, 1, 0\}$.

As shown in Fig AA, the input sequence is fed into the trained DARSI model to generate the value for $\vec{p}$. We can, therefore, write this forward pass of the sequence through the network as

$$\vec{p} = f(\mathbf{x}), \tag{S2}$$

where $\mathbf{x}$ represents the input sequence and $f(x) : \mathbb{R}^{4\times160} \rightarrow \mathbb{R}^3$ denotes all the layers of trained DARSI network, all lumped together in $f(\mathbf{x})$.

To compute the saliency map for the sequence image $\mathbf{x}$, we calculate the gradient of the loss function with respect to each position x_{ij} in the input sequence by applying the chain rule iteratively from the output layer to the input layer of the network such that

$$\frac{\partial L}{\partial x_{ij}} = \frac{\partial L}{\partial \vec{p}} \frac{\partial \vec{p}}{\partial f} \frac{\partial f}{\partial x_{ij}} \quad \forall i \in \{1, 2, \ldots, 160\} \quad \forall j \in \{1, 2, 3, 4\}. \tag{S3}$$

Because $f(\mathbf{x})$ consists of multiple layers and transformations, computing this derivative requires an iterative approach. Starting from the loss function, we iteratively propagate gradients backward through the network using the chain rule of differentiation, layer by layer, until the input is reached. This procedure, known as backpropagation, emphasizes the reverse traversal of layers to compute the necessary gradients.

To illustrate the computation of gradients through a convolutional layer, we present a detailed example. Consider a matrix $A \in \mathbb{R}^{2\times2}$ as the input to a two-dimensional convolutional layer to which a single filter $W \in \mathbb{R}^{1\times2}$ is applied, resulting in an output $y = f(A)$, as depicted in Fig AB. This example demonstrates the step-by-step process of propagating gradients through the convolutional layer.

First, the convolution operation is performed using a filter with known parameters $W = [w_1, w_2]$, that were obtained in the training process. The resulting output is transformed by applying a non-linearity, in this case, the logarithmic function. The output of the convolution is expressed as

$$A * W = \begin{pmatrix} \log(a_{11}w_1 + a_{12}w_2) & \log(a_{12}w_1 + a_{13}w_2) & \log(a_{13}w_1 + a_{14}w_2) \\ \log(a_{21}w_1 + a_{22}w_2) & \log(a_{22}w_1 + a_{23}w_2) & \log(a_{23}w_1 + a_{24}w_2) \end{pmatrix}. \tag{S4}$$

The final output of this convolutional layer is a non-linear transformation $y = q(A * W)$, where $q(.)$ denotes the logarithmic activation function. To compute the gradient of the output y with respect to a specific input element a_{ij}, we apply the chain rule of differentiation. For instance, the derivative with respect to a_{11} is given by

$$\frac{\partial y}{\partial a_{11}} = \begin{pmatrix} \frac{w_1}{a_{11}w_1+a_{12}w_2} & 0 & 0 \\ 0 & 0 & 0 \end{pmatrix} \tag{S5}$$

This calculation illustrates how convolutional layers integrate contributions from neighboring elements of the input matrix. For example, the value a_{12} contributes to the saliency computed for a_{11}, highlighting the localized yet interconnected nature of saliency computation in convolutional architectures. This same approach to backpropagation can be used to compute the saliency maps by propagating from the loss function as shown in Fig AA.

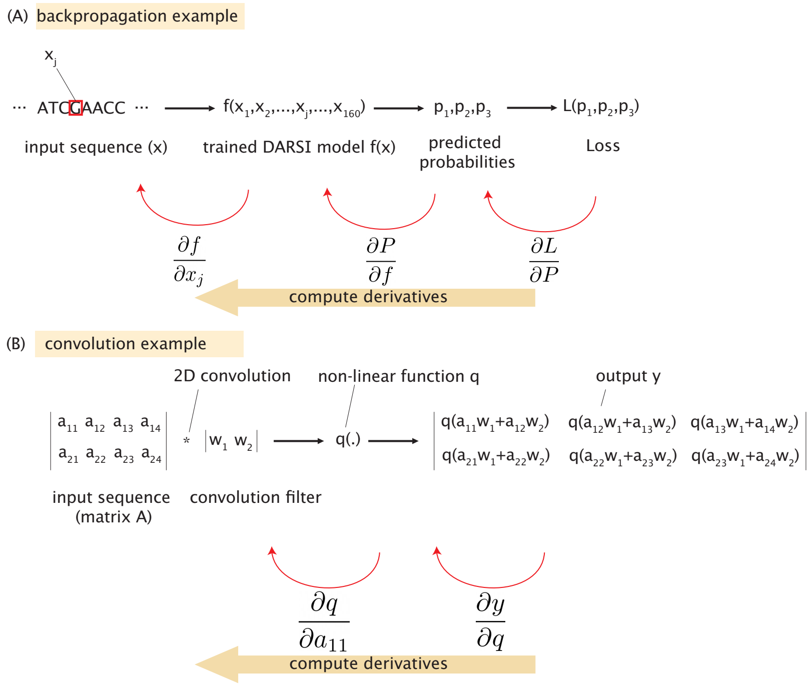

Fig A. Illustration of the backpropagation process and convolution operation in the DARSI model. (A) Example of backpropagation with an input DNA sequence processed by the trained DARSI model $f(x)$, which predicts probabilities p_1, p_2, p_3. The loss function $L(p_1, p_2, p_3)$ is computed using the probability vector $\vec{p} = [p_1, p_2, p_3]^T$. **(B)** Demonstration of a 2D convolution operation applied to a segment of the input sequence using a filter w_1, w_2, followed by a non-linear activation function $q(x) = \log x$.
