# Supplementary material for: Predictive modeling of gene expression and localization of DNA binding site using deep convolutional neural networks
Source: PLoS Comput Biol. 2026 Apr 1;22(4):e1014092. doi: 10.1371/journal.pcbi.1014092 (PMC13052891; doi:10.1371/journal.pcbi.1014092)
Supplement: S1 Fig — (PDF) [file pcbi.1014092.s009.pdf]

Supplemental Figures

Gene Expression Sensitivity to Mutation Plots

Examples of gene expression sensitivity plots for three illustrative operons along with cartoon of their inferred regulatory architecture is given in Fig A. Similar plots for all 95 operons can be found in the GitHub repository.

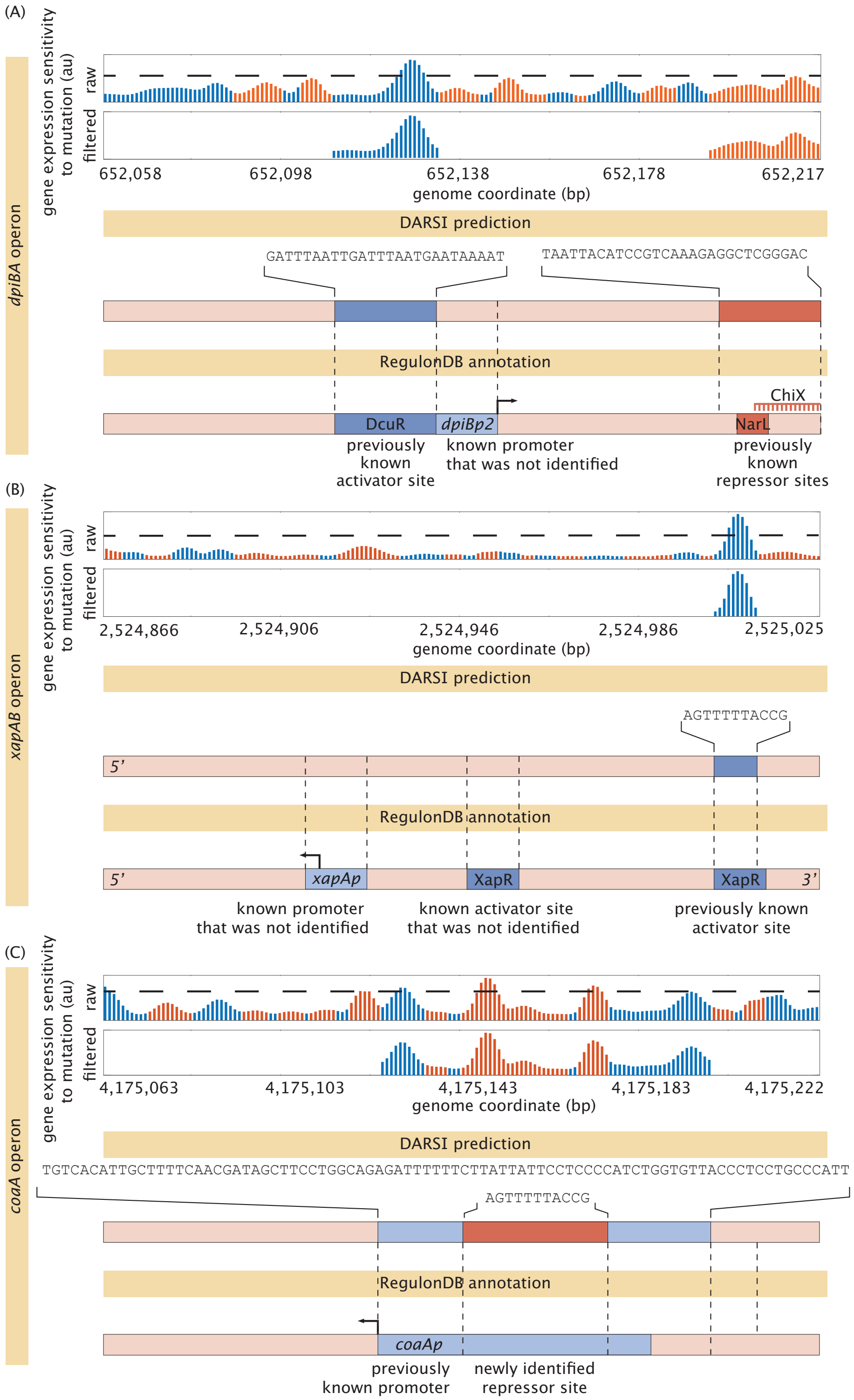

Fig A. Illustrative examples of expression sensitivity plots. Plots of raw expression sensitivity to mutation are presented for three illustrative operons selected to demonstrate distinct scenarios of the performance of DARSI. Each raw sensitivity plot is accompanied by its filtered version (obtained using the threshold indicated by the dashed line), which was used to infer the location and type of binding sites (activators vs. repressors). Additionally, regulatory cartoons depict the predicted binding sites, their sequences, and previous annotations based on RegulonDB (Tierrafria et al., 2022). It should be noted that the sequences are always presented in the 5' to 3' direction regardless of the strand. **(A)** Shows how DARSI successfully identified, as well as missed, previously annotated binding sites in the *dpiBA* operon. **(B)** While DARSI identified an already known site in the *xapAB* operon, it failed to another already known binding site as well as the promoter. **(C)** In the *coaA* operon, DARSI successfully identified the promoter as well as predicted a new repressor site.
