# Supplementary material for: Predictive modeling of gene expression and localization of DNA binding site using deep convolutional neural networks
Source: PLoS Comput Biol. 2026 Apr 1;22(4):e1014092. doi: 10.1371/journal.pcbi.1014092 (PMC13052891; doi:10.1371/journal.pcbi.1014092)
Supplement: S2 Fig — (PDF) [file pcbi.1014092.s010.pdf]

Supplemental Figures

Expression Plots Comparison

Fig A shows examples of unfiltered expression sensitivity-to-mutation plots generated by DARSI stacked against mutual information plots implemented as discussed in (Ireland et al. (2020)). Similar comparison plots for all 95 operons can be found in the GitHub repository.

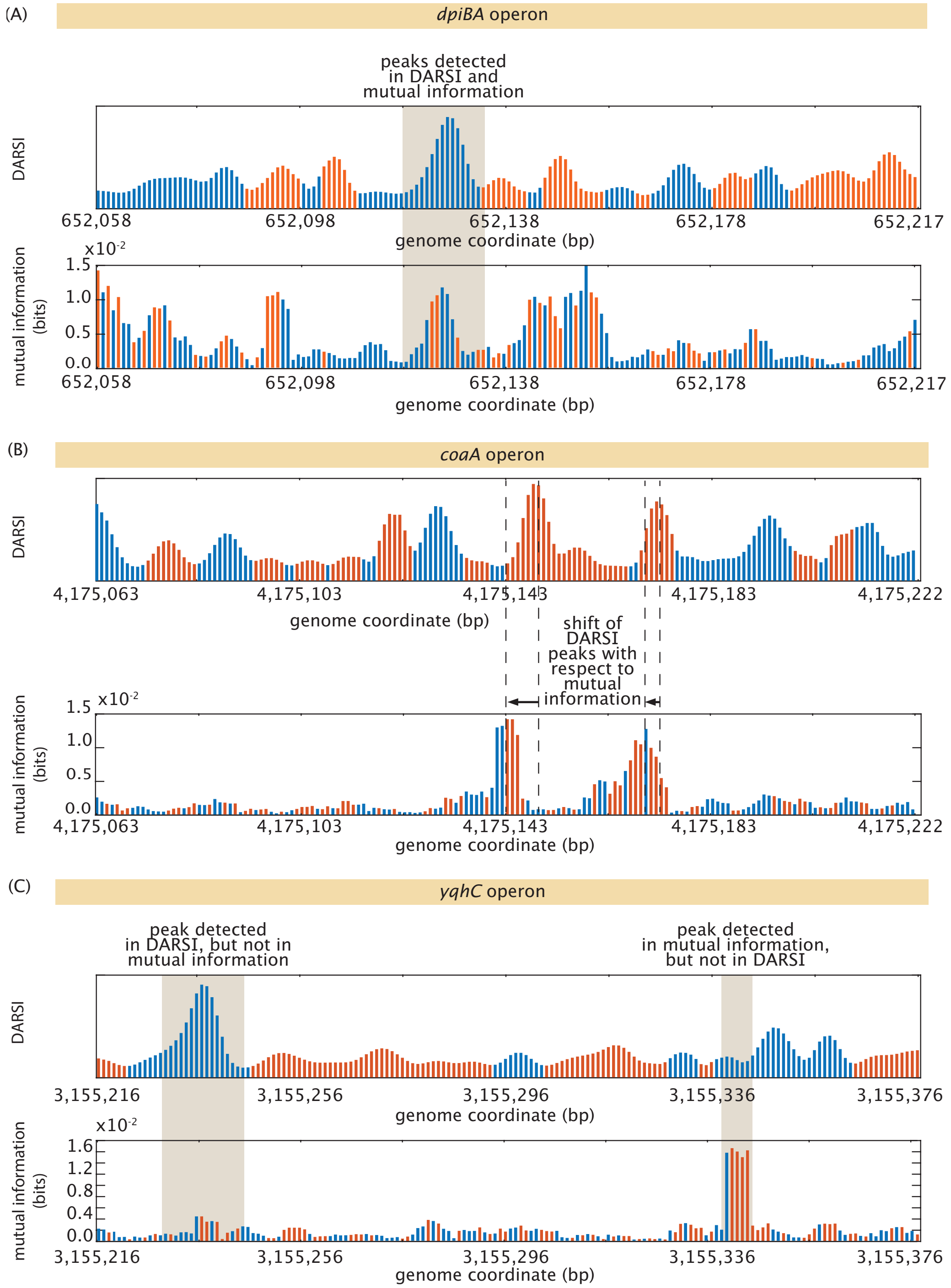

Fig A. Illustrative DARSI gene expression sensitivity plots compared with mutual information. Gene expression sensitivity-to-mutation plots generated by DARSI and mutual information for three illustrative operons. (A) The *dpiBA* exemplifies an agreement of some peaks detected by DARSI and by mutual information. (B) These peaks, however, can be displaced between these two measures as shown here for the *coaA* operon. (C) Analysis of the data for the *yqhC* operon reveals that peaks can be found by one measure but not the other one.
